# Cucurbitacin IIa Ameliorates DSS-Induced Ulcerative Colitis via Enhancing Intestinal Barrier Function and Inhibiting PERK/ATF4/CHOP Signaling Pathway

**DOI:** 10.5152/tjg.2024.24449

**Published:** 2024-12-23

**Authors:** Yang Yang, Qiong Yang

**Affiliations:** Wuxi No. 2 People’s Hospital, Wuxi, China

**Keywords:** Cucurbitacin IIa, ulcerative colitis, intestinal barrier function, PERK/ATF4/CHOP signaling pathway, inflammatory factors

## Abstract

**Background/Aims:**

The primary intent of this manuscript is to ascertain the effect of cucurbitacin IIa on ulcerative colitis (UC) and illustrate the potential mechanisms based on intestinal barrier function and the PERK/ATF4/CHOP signaling pathway.

**Materials and Methods:**

The UC mouse model was constructed by drinking 3% dextran sulfate sodium (DSS) for 1 week. The colonic tissues were stained with HE to assess pathological changes. The enzyme linked immunosorbent assay was used to measure myeloperoxidase (MPO) activity and levels of IL-1β, IL-6, and TNF-α. The western blot and immunohistochemistry methods were performed to analyze the expressions of PERK/ATF4/CHOP pathway-associated proteins. The quantitative real-time polymerase chain reaction (qRT-PCR) and immunofluorescence analysis were carried out to determine the expressions of ZO-1, claudin-1, occludin mRNA, and protein.

**Results:**

In comparison with the model group, cucurbitacin IIa obviously increased body weight and colon length, reduced disease activity index value and MPO activity, and ameliorated the degree of histopathological damage. Inflammatory factor levels were considerably reduced in the cucurbitacin IIa-intervention groups compared to the model group. The western blot and immunohistochemistry results indicated that, compared with the model group, cucurbitacin IIa significantly abolished the protein expressions of p-PERK, p-eIF2α, ATF4, and CHOP. The results of qRT-PCR and immunofluorescence revealed that cucurbitacin IIa greatly elevated the expressions of ZO-1, claudin-1, occludin mRNA and protein.

**Conclusion:**

Cucurbitacin IIa dramatically ameliorates DSS-induced UC symptoms via suppressing the PERK/ATF4/CHOP pathway and reinforcing enteric barrier function.

**Main Points**

- Cucurbitacin IIa ameliorates UC.
- Cucurbitacin IIa enhances the intestinal barrier function of UC mice.
- Cucurbitacin IIa inhibits the PERK/ATF4/CHOP signaling pathway of UC mice.

## Introduction

Ulcerative colitis (UC) is a long-term, nonspecific inflammatory disease whose etiology is still not very clear. The lesions are mostly located in the sigmoid colon and rectum but can also extend to the descending colon or even the whole colon, with a long course of the disease, often with recurring episodes.^1^ Clinical manifestations include diarrhea, mucopurulent blood stools, and abdominal pain. The severity of the disease varies, and most cases have a recurrent chronic course. The disease can occur at any age but is mostly 20-40 years old. It can also be observed in children or the elderly. There is no significant difference in incidence between men and women. Generally, the incidence of this disease is relatively high in Western countries. Commonly used therapeutic drugs include aminosalicylic acid preparations, glucocorticoids, immunosuppressive agents, and biological agents.^2^ Surgery may be required when complications such as hemorrhage, perforation, toxic megacolon, and carcinoma occur. There are some defects or shortcomings in the current treatment methods. For example, some patients may not be sensitive or resistant to drug treatment, and long-term use of drugs may bring some adverse reactions. Although surgical treatment can resolve some serious complications, the surgery itself has certain risks and complications and may affect intestinal function after the operation.^3^ There is an urgent need to find safe and effective drugs to treat UC.

**Figure 1.**
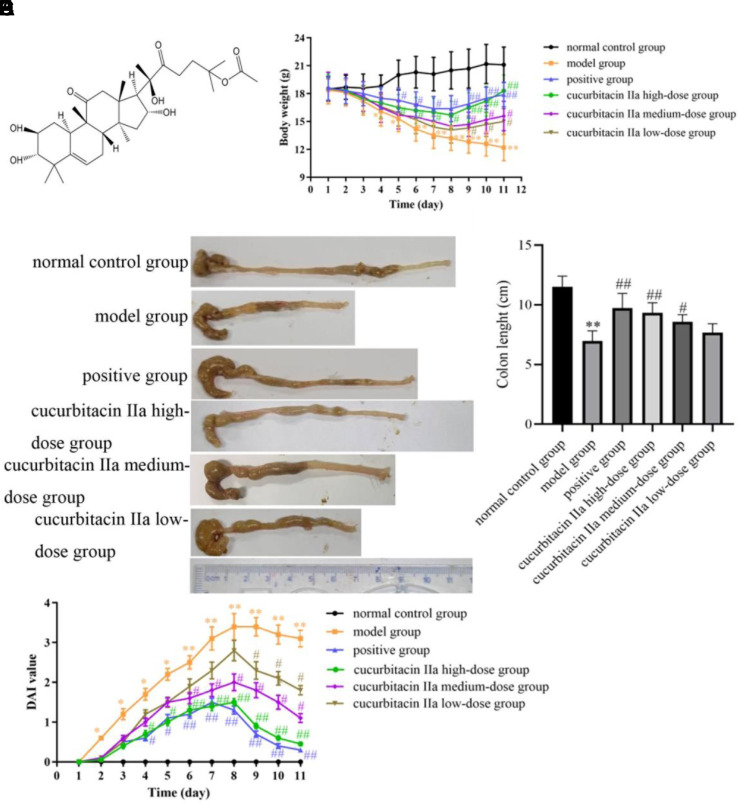
Cucurbitacin IIa alleviated the symptoms of UC. (A) The structure of cucurbitacin IIa. (B) Effect of cucurbitacin IIa on the body weight. (C, D) Effect of cucurbitacin IIa on colon length. (E) Effect of cucurbitacin IIa on the DAI value. Data were expressed as mean ± SD (n = 10). ^**^P < .05, ^**^P < .01 versus normal control group; ^#^*P* < .05, ^##^*P* < .01 versus model group.

Cucurbitacin IIa (C_32_H_50_O_8_, 23,24-dihydrocucurbitacin F-25-*O*-acetate) belongs to the tetracyclic triterpenoid (Figure 1A), which is found in plants such as *Hemsleya*.^4^ Cucurbitacin IIa is soluble in organic solvents, such as petroleum ether, chloroform, and dichloromethane, and insoluble in water. Cucurbitacin IIa possesses multiple bioactivities, including anti-allergic, anti-inflammatory, anti-cancer, human immunodeficiency virus inhibition, antidepressant, and so on.^5-8^ Cucurbitacin IIa exerts these pharmacological effects by modulating various signaling and metabolic pathways. However, it has not been reported in the literature whether cucurbitacin IIa could be used for the therapeutic management of UC and the underlying potential mechanism of action.

Intestinal barrier function is strongly implicated in the evolution of UC.^9^ When the intestinal barrier function is impaired, such as through the disruption of the intestinal epithelial tight junctions and thinning of the mucus layer, it increases intestinal permeability. This makes it easier for bacteria, toxins, and other harmful substances in the intestines to cross the intestinal mucosa into the lamina propria, triggering abnormal immune responses and inflammation. These inflammations continue to irritate and damage the intestinal mucosa, leading to the development and progression of UC.^10,11^ During the course of UC, the inflammatory response itself will further aggravate the destruction of the intestinal barrier, forming a vicious cycle. Studies have found that endoplasmic reticulum (ER) stress may trigger the unfolded protein response (UPR), which activates the caspase-12 pathway, inducing apoptosis of intestinal epithelial cells, thus impairing intestinal barrier function.^12^ In addition, ER stress also affects the balance of intestinal flora, and the imbalance of intestinal flora further damages the integrity of the intestinal barrier.^13^ PERK/ATF4/CHOP is an important signaling pathway associated with ER stress.^14,15^ Previous studies have found that the PERK/ATF4/CHOP pathway was elevated in the bowel tissue of UC patients and correlated with disease severity. Inhibition of the PERK/ATF4/CHOP signaling pathway can reduce intestinal inflammation and tissue damage and improve symptoms of UC.^16,17^

In the present investigation, we evaluated the effectiveness of cucurbitacin IIa in the treatment of UC mice and explored its effects on PERK/ATF4/CHOP and intestinal barrier function.

## Materials and Methods

### Animals and Reagents

SPF-grade male C57BL/6J mice (20 ± 2 g, 6-7 weeks old) were acquired from Sibeifu (Suzhou) Biotechnology Co., LTD., under certificate no. SCXK (Su) 2022-0006. The animals were housed at room temperature (25.0 ± 0.5)°C, relative humidity (55 ± 5)%, and natural day-light/dim cycle. Cucurbitacin IIa and mesalazine were purchased fromSigma-Aldrich Chemical Co. (St. Louis, Missouri, USA) . Dextran sulfate sodium (DSS, 36 000-50 000 Da) was purchased from MP Biomeicals (OH, USA). Mouse IL-1β, IL-6, TNF-α, and myeloperoxidase (MPO) enzyme-linked immunosorbent assay (ELISA) kits were acquired from Jianglai Biological Co. (Shanghai, China). Antibodies against PERK, p-PERK, eIF2α, p-eIF2α, ATF4, CHOP, claudin-1, occludin, ZO-1, and β-actin were received from Cell Signaling Technology (Danvers, MA, USA).

### Induction of UC Mouse Model, Drug Intervention, and Sample Collection

Animal experiments were approved by the Laboratory Animal Ethics Committee of Wuxi No.2 People’s Hospital (approval number: 2024-009; date: 16 January, 2024), and conducted under its guidelines. After a 1-week adaptation, C57BL/6J male mice were stochastically categorized into 6 groups (n = 10): normal control group, model group, positive group (mesalazine, 100 mg/kg), and cucurbitacin IIa high-, medium-, and low-dose groups. According to the pre-experimental data, the dosage of cucurbitacin IIa in the high-, medium-, and low-dose groups was 120, 60, and 30 mg/kg, respectively. The mice in the normal control group were given normal saline for 7 days, and the mice in the other groups were freely given 3% DSS solution for 7 days to establish an acute colitis mouse model. The mice in the positive group, cucurbitacin IIa high-, medium-, and low-dose groups, received the corresponding doses of drugs by intragastric administration from days 1 to 10. Following the final gavage, all animals were abstained from food for 24 hours, anesthetized by isoflurane inhalation. Blood was removed from the eyeballs, and the mice were then sacrificed by decervicalization. The length of the intact colon was gauged and photographed. One part of the colon was stored in 4% paraformaldehyde and the other part was stored in a −80°C fridge for subsequent studies.

### Disease Activity Index Score

Observations and records were made on weight, diarrhea, blood in stool, and activity status. The scoring criteria of the disease activity index (DAI) of colitis mice are shown in Table 1. DAI = (body mass loss score + diarrhea score + blood in stool score)/3.^18^

### Detection of Colon Length and Histological Evaluation

The mice were anesthetized and euthanized, dissected to remove the colon, and the colon length was measured and photographed. Part of the colon tissue was immobilized with 4% paraformaldehyde, dewatered, blocked with paraffin, and chopped into 5 μm slices for hematoxylin-eosin (HE) staining. The tissue morphology was observed under a light microscope and histopathological scoring was performed according to the scoring method of Almarzooqi et al.^19^ The criteria of the histological score are summarized in Table 2.

### Enzyme-Linked Immunosorbent Assay Methods for Inflammatory Factors and MPO Values

The blood of each group was successfully separated by centrifugation at 4°C, 3500 r/min for 15 minutes, and the uppermost layer of serum was obtained. The IL-1β, IL-6, TNF-α levels in the serum and colonic tissues, and MPO activity in the colonic tissues were measured following the specifications of ELISA kits.

### Expression of PERK/ATF4/CHOP Pathway-Related Proteins in Colon Tissues by Western Blot Methods

An appropriate amount of colon tissue was taken and placed in a pre-cooled homogenizer. Lysate was added to fully homogenize the tissue, and the supernatant was collected by centrifugation after a period of time on ice. Then, the tissue protein extract was obtained. The protein levels were precisely calculated using a protein quantification kit. Based on the amount of protein, an appropriate amount of sample was taken and mixed with the sampling buffer, and then subjected to sodium dodecyl sulfate-polyacrylamide gel electrophoresis (SDS-PAGE). After electrophoresis, the proteins in the gel were transferred to a polyvinylidene fluoride membrane. Following the covering the membrane with 5% skimmed milk powder for about 2 hours, primary antibodies were incubated for 12 hours at 4°C. The subsequent day, excess primary antibody was washed away, secondary antibody was introduced and cultured for 1.5 hours at ambient temperature. After sufficient washing, enhanced chemiluminescence (ECL) reagent was used to develop the color, and a chemiluminescence imaging system was used to obtain the images and analyze the gray value of the protein bands, so as to determine the expressions of these related proteins in the colon tissue.

### Expression of PERK/ATF4/CHOP Pathway-Related Proteins in Colon Tissues Detected by Immunohistochemical Methods

Mouse colon tissues of 1 cm length were taken from each group, rinsed with saline, immobilized in 4% paraformaldehyde solution, desiccated in graduated ethyl alcohol, clarified, wax-dipped, paraffin-embedded, sectioned, and baked, then routinely deparaffinized with xylene, hydrolyzed in graduated alcohols, incubated with 3% BSA (Bovine Serum Albumin) at ambient temperature for 0.5 hours, inserted with primary antibodies (PERK, p-PERK, eIF2α, p-eIF2α, ATF4, and CHOP), and incubated at 4°C overnight. Sections were irrigated 3 times with phosphate-buffered saline (PBS) for 5 minutes each time. After adding the secondary antibody, the incubation was carried out at 37°C for 30 minutes. The slices were irrigated with PBS 3 times, and then incubated with DAB (3,3'-Diaminobenzidine), washed with water, and colored with hematoxylin. The slices were routinely dehydrated, cleared, and blocked with neutral gum. The samples were visualized under a microscope and photos were taken.

### Quantitative Real-Time Polymerase Chain Reaction Analysis

Thirty mg of colon tissue was weighed into a grinding tube, then the Trizol method was performed to extract RNA, followed by denaturation and cooling. The RNA was back-transcribed into cDNA in accordance with the description of the reverse transcription amplification kit, and the requirements for the reverse transcription reaction were as described below: 37°C for 15 minutes (reverse transcription reaction), 85°C for 5 seconds (inactivation of the reverse transcriptase reaction), and 4°C. According to the instructions of the quantitative polymerase chain reaction (PCR) kit, the reverse transcription reaction solution was added to the PCR reactor for annealing, extension, and inactivation, and the PCR reaction conditions were as follows: pre-denaturation at 95°C for 10 minutes, 95°C for 10 seconds, 55°C for 20 seconds, 72°C for 40 seconds, 40 cycles. The 2^−ΔΔCt^ approach was applied to quantify the subject gene, and β-actin was employed as an in-parameter. The employed primer sequences are listed as follows: ZO-1 forward primer: 5’-TAAAGCTGTCCCTGTGAGTCCTTC-3’; ZO-1 reverse primer, 5’-TCTATGGAACTCAACACACCACCA-3’; claudin-1 forward primer, 5’-AGTCTTCGACTCCTTGCTGAATCT-3’; claudin-1 reverse primer, 5’-ATCCACATCTTCTGCACCTCATCA-3’; occludin forward primer, 5’-CTCCTCCAATGGCAAAGTGAATGG-3’; occludin reverse primer, 5’-CATCCACACTCAAGGTCAGAGGAA-3’; β-actin forward primer, 5’-CAACGGCTCCGGCATGTG-3’; β-actin reverse primer, 5’-AGTCCTTCTGACCCATTCCCA-3’.

### Immunofluorescence Analysis of Tight Junction Proteins

Paraffin-embedded tissues were cut into approximately 5 μm thick slices using a sectioning machine. The processed tissue sections were incubated with antibodies against ZO-1, claudin-1, and occludin for 12 hours at 4°C, afterward washed with PBS for 5 minutes each time for a total of 3 times. Fluorescein isothiocyanate (FITC)-conjugated goat anti-rabbit IgG was inserted and conjugated for 1 hour in a dimmed chamber. The sections were then removed and processed with PBS 3 times, and then blocked for fluorescence observation under a microscope.

### Statistical Analysis

Statistical analyses were undertaken using SPSS 26.0 software (IBM SPSS Corp.; Armonk, NY, USA), and values were expressed as mean ± SD. Between-group comparisons were visualized using analysis of variance (one-way ANOVA), and *P* < .05 was considered to be statistically meaningful.

## Results

### Effect of Cucurbitacin IIa on General Status, Body Weight, Colon Length, and DAI Value in Mice with UC

In comparison to the normal control group, the mice in the model group experienced changes such as lethargy, piling up, and disorganized hair near the anus on the third day of modeling, and then gradually worsened with the phenomenon of blood in the stool from the fourth to 11th day. Additionally, the body weight gradually decreased from the second day, with a significant difference observed from the fourth day onward (*P* < .05, *P* < .01, Figure 1B). Compared to the normal control group, the colon length of mice in the model group was markedly reduced (*P* < .01, Figure 1C and D), and the DAI value in the model group was remarkably escalated from the second day (*P* < .05, *P* < .01, Figure 1E). However, the positive group (from fifth day to 11th day), cucurbitacin IIa high-dose group (from fifth day to 11th day), medium-dose group (from ninth day to 11th day), and low-dose group (from ninth day to 11th day) significantly increased the body weight in the model group (*P* < .05, *P* < .01). The colon length in the positive group, cucurbitacin IIa high-dose and medium-dose groups was considerably longer than that in the model group (*P* < .05, *P* < .01). The colon length in the cucurbitacin IIa low-dose group was longer than that in the model group, but without meaningful variation (*P >* .05). The DAI value in the positive group (from fourth day to 11th day), cucurbitacin IIa high-dose group (from fourth day to 11th day), medium-dose group (from sixth day to 11th day), and low-dose group (from ninth day to 11th day) was lower than that in the model group (*P* < .05, *P* < .01).

### Effect of Cucurbitacin IIa on Colonic Pathologic Changes and MPO Activity in Mice with UC

**Figure 2.**
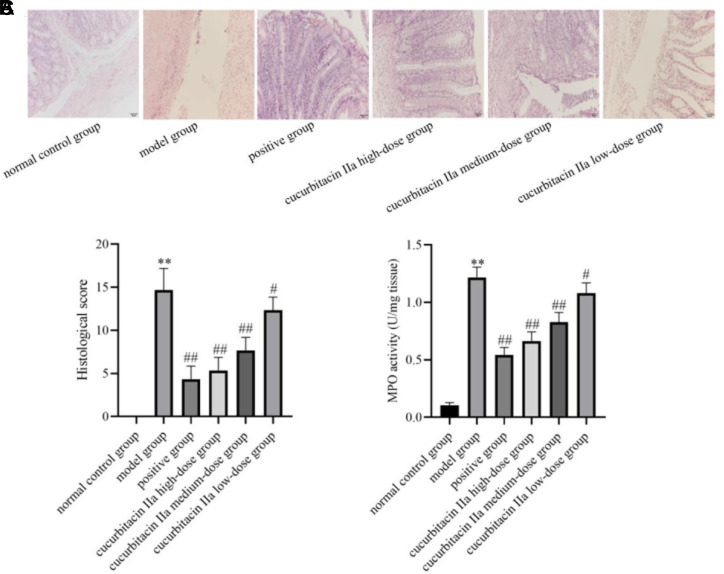
Cucurbitacin IIa reduced the colonic histopathological damage and MPO activity in mice with UC. (A) HE staining. (B) Histological score. (C) MPO activity. Data were expressed as mean ± SD (n = 3). ^**^P < .01 versus normal control group; ^#^*P* < .05, ^##^*P* < .01 versus model group.

In comparison to the normal control group, HE staining in the model group displayed a greater infiltration of inflammatory cells in the colonic tissue, and more cup and crypt cells disappeared. The mucosal tissue structure was disorganized, and the pathological damage score of the colonic tissues was significantly elevated (*P* < .01, Figure 2A and B). The positive group, cucurbitacin IIa high-, medium- and low-dose groups resulted in decreased colonic tissue damage, decreased congestion and infiltration of inflammatory cells in the lamina propria of the colon, a significant increase in the number of cup cells and crypt cells, and a significant decrease in the colonic histopathological damage score when compared with the model group (*P* < .01). The MPO activity in the model group was significantly higher than that in the normal control group (*P* < .01, Figure 2C). However, the positive group, cucurbitacin IIa high-, medium- and low-dose groups remarkably reduced the MPO activity in the model group (*P* < .05, *P* < .01).

### Effects of Cucurbitine IIa on Inflammation in UC Mice

**Figure 3.**
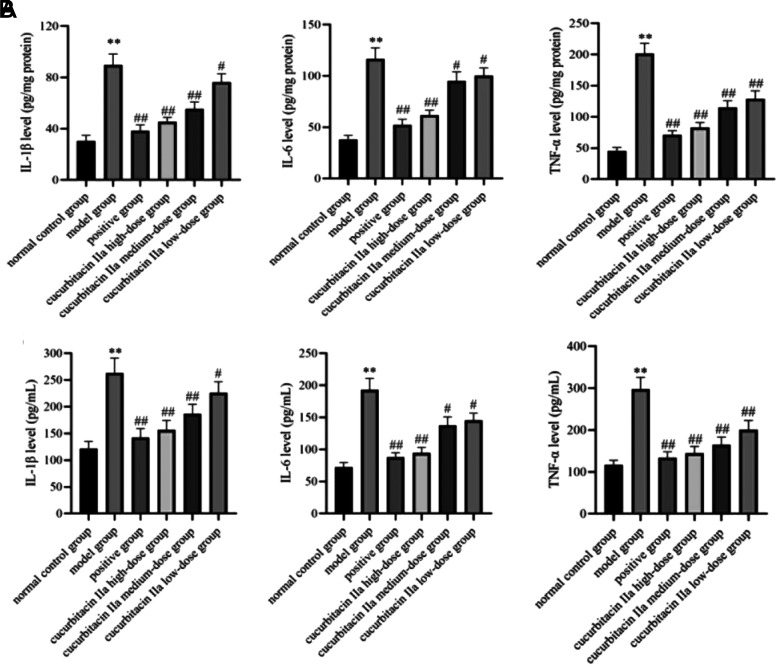
Effect of cucurbitacin IIa on inflammatory factors in the colonic tissue and serum of mice with UC. (A) Colonic tissue. (B) Serum. Data were expressed as mean ± SD (n = 3). ^**^P < .01 versus normal control group; ^#^*P* < .05, ^##^*P* < .01 versus model group.

The levels of IL-1β, IL-6, and TNF-α in the colon and serum of the model group mice were significantly higher than those in the normal control group (*P* < .01, Figure 3). When compared with the model group, the content of IL-1β, IL-6, and TNF-α in the colon and serum of mice was significantly decreased in the positive group and the cucurbitacin IIa high-, medium- and low-dose groups (*P* < .05, *P* < .01).

### Effect of Cucurbitacin IIa on PERK/ATF4/CHOP Signaling Pathway

**Figure 4.**
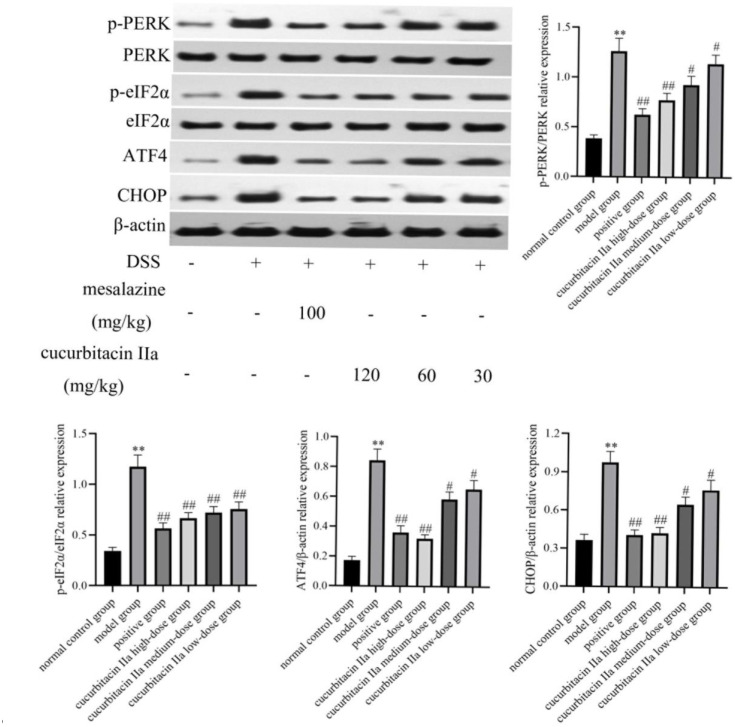
Western blot analysis and quantitative analysis of PERK/ATF4/CHOP signaling pathway-associated protein expressions. Data were expressed as mean ± SD (n = 3). ^**^P < .01 versus normal control group; ^#^*P* < .05, ^##^*P* < .01 versus model group.

**Figure 5.**
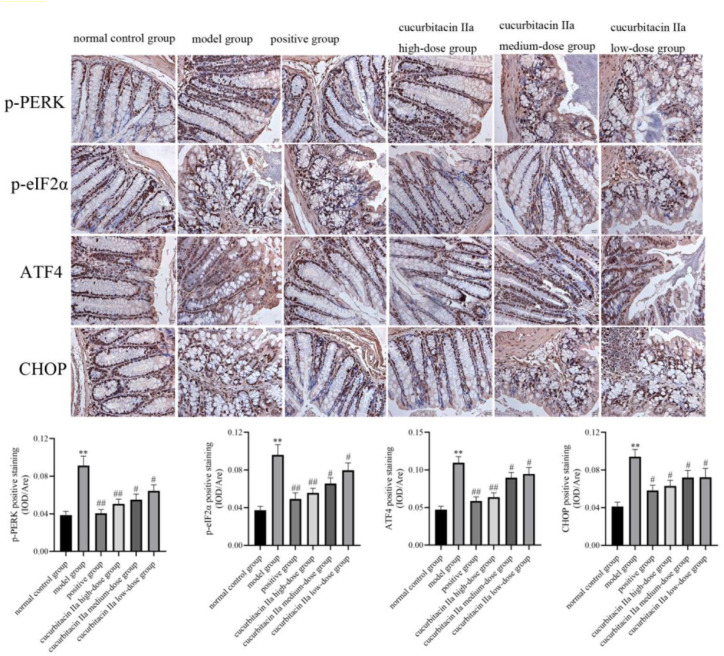
Immunohistochemistry analysis and quantitative analysis of p-PERK, p-eIF2α, ATF4, CHOP proteins. Data were expressed as mean ± SD (n = 3). ^**^P < .01 versus normal control group; ^#^*P* < .05, ^##^*P* < .01 versus model group.

As shown in Figure 4, western blot analysis indicated that compared with the normal control group, PERK was activated by DSS administration via strengthening its phosphatization in colonic tissues (*P* < .05, *P* < .01). Additionally, the PERK pathway-associated proteins, such as p-eIF2α, ATF4, and CHOP were significantly enhanced by DSS stimulation. However, cucurbitacin IIa treatment restrained the PERK/ATF4/CHOP signaling pathway. Likewise, the immunohistochemistry analysis showed that the brown staining of p-PERK was enhanced in the model group when compared with that in the normal control group (*P* < .05, Figure 5). However, cucurbitacin IIa treatment reversed the increased brown staining in the model group. Similar trends can be observed in p-eIF2α, ATF4, and CHOP proteins. These results confirmed that cucurbitacin IIa relieved UC in mice via suppressed PERK/ATF4/CHOP signaling pathway.

### Effect of Cucurbitacin IIa on Intestinal Barrier Function

**Figure 6.**
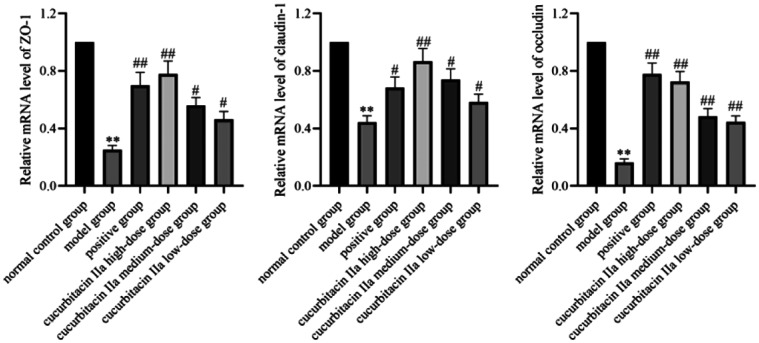
The mRNA levels of ZO-1, claudin, and occludin in colonic tissues. Data were expressed as mean ± SD (n = 3). ^**^P < .01 versus normal control group; ^#^*P* < .05, ^##^*P* < .01 versus model group.

**Figure 7.**
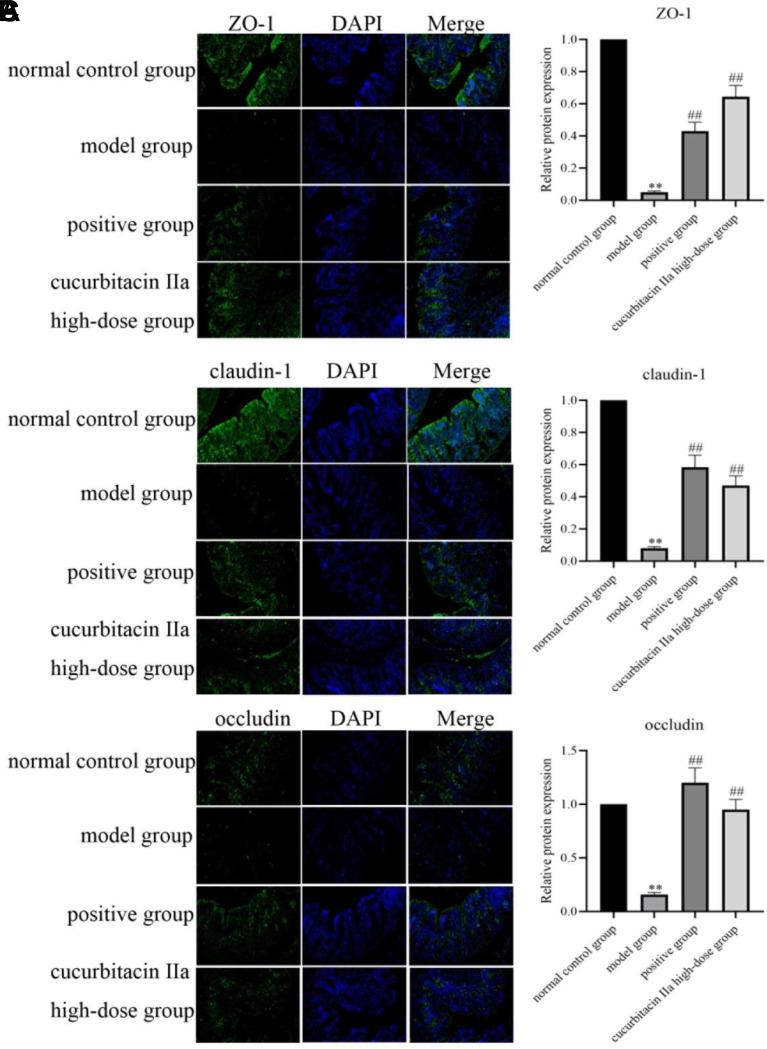
The expressions of colonic tight-junction proteins were detected by immunofluorescence analysis and its quantification analysis. (A) ZO-1. (B) Claudin. (C) Occludin. Data were expressed as mean ± SD (n = 3). ^**^P < .01 versus normal control group; ^##^*P* < .01 versus model group.

In the present study, the mRNA levels of ZO-1, claudin-1 and occludin in the model group were lower than those in the normal control group (*P* < 0.01, Figure 6). However, the positive group and cucurbitacin IIa intervention groups remarkably enhanced the mRNA levels of the above 3 tight junction proteins (*P* < .05, *P* < .01). Meanwhile, compared to the normal control group, immunofluorescence analysis revealed that DSS induction in mice reduced the protein expressions of ZO-1, claudin-1 and occludin (*P* < .01, Figure 7). However, we found a pronounced increase in these tight junction protein expressions in the positive group and cucurbitacin IIa high-dose group (*P* < .01). The aforementioned outcomes were indicative of the fact that cucurbitacin IIa enhanced intestinal barrier function in UC mice.

## Discussion

The pathogenesis of UC is complex and involves the interaction of multiple factors, including genetic factors, intestinal microecological imbalance, abnormal nominal function, and the environment. If left untreated, it can lead to the widening of lesions, severe diarrhea and pus-blood stools, intestinal complications (e.g., intestinal perforation, toxic megacolon), intestinal stenosis, and increased risk of colon cancer. Treatment includes medication and surgery, along with rest and dietary modifications. The current treatment of UC has some of the following shortcomings or challenges: (1) Inability to cure: most of the existing treatments can only control the disease and maintain remission, but it is difficult to completely cure the disease, and patients often need long-term treatment or even life-long treatment. (2) Drug side effects: some drugs such as glucocorticoids have many side effects with long-term use, such as osteoporosis, elevated blood glucose, increased risk of infection, etc.; immunosuppressive drugs bring multiple undesirable consequences, such as osteoporosis, elevated blood glucose, higher risk of developing serious infections, etc.; immunosuppressants have an increased risk of cancer and myelosuppression. (3) Individual differences: the response of different patients to drugs varies greatly, and some patients are insensitive or resistant to conventional treatments, so they need to try different drug combinations and regimens. (4) Cost of treatment: long-term medication and possible repeated visits to the doctor will bring a certain economic burden. (5) Lack of precise treatment: although research into the pathogenesis is constantly deepening, there is a lack of precise treatment for the disease, but it is not easy to find a solution. Despite deepening research into the mechanism, completely precise individualized treatment has not yet been achieved, and the choice of treatment plan still relies on experience to a certain extent. (6) Difficulty in the treatment of extra-intestinal manifestations: for some accompanying extra-intestinal manifestations, such as arthritis and ocular lesions, the treatment effect may be unsatisfactory. (7) Impact in terms of human life: although the disease is under control, it may still have a continuous negative impact on the daily life of the patient, their work, and their psychological state. etc., causing continuous negative impact.^20-22^ Therefore, it is essential to explore new potential therapeutic interventions for patients with UC who have failed treatment with traditional methods.

Cucurbitacin IIa, a member of the cucurbitacin family, isolated exclusively from the roots of the *Hemsleya* plant, is the main ingredient in the Chinese prescription Hemslecin capsules and tablets.^23^ Cucurbitacin IIa has been used as an ancient remedy for bacillary dysentery and gastroenteritis. Cucurbitacin IIa, as a natural compound with better safety and tolerability, has significant advantages in clinical applications and exerts anti-inflammatory effects through a variety of pathways, with broader modulatory capabilities in inflammatory-related diseases.^8,24,25^ Although the anti-inflammatory effects of cucurbitacin IIa have been well-recognized for a long time, the protective effect of cucurbitacin IIa against UC remains unreported. In this research, we studied the amelioration of cucurbitacin IIa on DSS-induced UC and exploited its prospective mechanism of action. The outcomes demonstrated that cucurbitacin IIa alleviated UC by restraining PERK–ATF4–CHOP signaling pathway and restoring intestinal barrier function.

The pathogenesis of UC is intimately connected to the PERK–ATF4–CHOP signaling pathway.^16,17^ PERK is activated when intestinal cells are subjected to various stressful stimuli, and PERK activation phosphorylates eIF2α, causing repression of protein translation initiation and, at the same time, promotes the expression of ATF4, which further induces the expression of CHOP.^26,27^ The upregulation of CHOP can mediate inflammatory responses and apoptosis through various mechanisms, exacerbating intestinal tissue damage.^28^ The enteric barrier function consists of 4 components: physical, immune, biological, and chemical barriers.^29^ Among them, the mechanical barrier consists of enteric epithelial cells and inter-apical cellular tight junctions in the bowel mucosa. Studies have shown that the enhancement of the PERK–-ATF4–CHOP pathway disrupts the intestinal barrier function and leads to increased intestinal permeability, thereby promoting the development of inflammatory bowel disease and other intestinal-related diseases.^30,31^ The specific manifestations are: (1) affecting tight junction proteins: the PERK–ATF4–CHOP signaling pathway may affect tight junction proteins, e.g., claudin and occludin, thereby disrupting the completeness of the enteric mechanical barrier and increasing the permeability of the intestinal tract. (2) Inducing apoptosis: the upregulation of CHOP mediates inflammatory response and apoptosis through various mechanisms, aggravating intestinal tissue damage and dysfunction, and thus promoting the pathological process of UC.^17,32^ In this study, we found that cucurbitacin IIa notably reduced DSS-induced PERK phosphorylation. Meanwhile, the expressions of PERK downstream targeted proteins were significantly enhanced by DSS stimulation. However, cucurbitacin IIa effectively suppressed the above protein expressions. Our results enhanced the plausibility of cucurbitacin IIa for the treatment of UC through inhibition of the ER-stressed PERK–ATF4–CHOP pathway.

Ulcerative colitis is a continuous, diffuse inflammatory disease that occurs in the mucosa and submucosa of the colorectum. In this study, cucurbitacin IIa reduced symptoms of UC, including increasing body weight and colon length, reducing DAI value, as well as ameliorating histopathologic changes in the colon by decreasing congestion and infiltration of inflammatory cells and increasing the amount of cupped and crypt cells. In the mucous membranes of the colon from UC patients, neutrophil infiltration is one of the pathologic features. Myeloperoxidase is mainly found in neutrophils, and when neutrophils are recruited to the site of inflammation (e.g., the colonic mucosa of UC patients), MPO will be released into the extracellular environment accordingly. Therefore, the measurement of MPO activity can indirectly reflect the degree of neutrophil infiltration in the colonic mucosa, which in turn reflects the severity of inflammation in UC.^33^ The impaired mucosal barrier function is an implicated part of the pathogenesis of UC, and the oxidized products produced by MPO further aggravate the disruption of the mucosal barrier. A normal colonic mucosal barrier can block the migration of harmful substances, like bacteria and toxins, into the submucosa and the bloodstream. However, oxidative damage caused by elevated MPO activity will destabilize the compact tight junctions between intestinal epithelial cells, increase enteric permeability, and allow more harmful substances to enter the submucosa, thereby exacerbating the inflammatory response and creating a vicious cycle.^34^ In this study, the MPO activity in the cucurbitacin IIa intervention groups was weaker than that in the model group, consistent with the results that cucurbitacin IIa attenuates colonic tissue damage. In addition, cucurbitacin IIa decreased the higher levels of IL-1β, IL-6, and TNF-α in the model group. These results indicated that cucurbitacin IIa possessed anti-inflammatory activity and was able to alleviate the symptoms of DSS-induced UC in mice.

In conclusion, the presented study confirmed the amelioration of cucurbitacin IIa on DSS-induced UC. We further demonstrated that the mechanism of action of cucurbitacin IIa to protect UC may be through inhibiting the PERK–ATF4–CHOP signaling pathway of ER stress. Cucurbitacin IIa inhibited the inflammatory factor levels and enhanced the intestinal barrier function in mice with UC. Cucurbitacin IIa may be developed as a future drug equivalent for the management of UC, especially UC that is not amenable to current drug therapies.

## Figures and Tables

**Table 1.** DAI Scoring Criteria for DSS Mice

Scoring	Rate of Body Mass Loss (%)	Fecal Character	Hematochezia
0	No	Normal	Negative
1	1-5	Soft, formed stools	Occult blood (+)
2	6-10	Mucus, formed stools	Occult blood (++)
3	11-15	Soft, mucus, formed stools	Occult blood (+++)
4	>15	Soft, mucus, unformed stools	Massive bloody stool

**Table 2.** The Histological Score Criteria

Index	Scoring	Characteristic
Lesion range (%)	0	0%
	1	1%-25%
	2	26%-50%
	3	51%-75%
	4	76%-100%
Lesion depth	0	No lesion
	1	Mucosal epithelium
	2	Mucoderm
	3	Submucosal layer
	4	Muscular layer and serous layer
Crypt damage	0	Complete crypts
	1	Lost a third of the foundation
	2	Lost two-thirds of the foundation
	3	Lost the whole crypt and complete epithelial superficiality
	4	Double loss of crypt changes
Severity of inflammation	0	No inflammation
	1	Light leukocyte infiltration
	2	A small amount of leukocyte infiltration
	3	Moderate amount of leukocyte infiltration
	4	Massive leukocyte infiltration
Fibrous tissue proliferation	0	No fibrosis
	1	Light fibrosis
	2	A small amount of fibrosis
	3	Moderate amount of fibrosis
	4	Massive fibrosis

## Data Availability

The data that support the findings of this study are available on request from the corresponding author.
